# Ab Initio Accuracy Neural Network Potential for Drug-Like Molecules

**DOI:** 10.34133/research.0837

**Published:** 2025-08-25

**Authors:** Manyi Yang, Duo Zhang, Xinyan Wang, BoWen Li, Linfeng Zhang, Weinan E, Tong Zhu, Han Wang

**Affiliations:** ^1^ The Institute of Green Chemistry and Engineering, Nanjing University, Suzhou, Jiangsu 215163, China.; ^2^ AI for Science Institute, Beijing 100080, China.; ^3^ DP Technology, Beijing 100080, China.; ^4^ Shanghai Engineering Research Center of Molecular Therapeutics & New Drug Development, School of Chemistry and Molecular Engineering, East China Normal University, Shanghai 200062, China.; ^5^ Center for Machine Learning Research, Peking University, Beijing 100871, China.; ^6^ School of Mathematical Sciences, Peking University, Beijing 100871, China.; ^7^ National Key Laboratory of Computational Physics, Institute of Applied Physics and Computational Mathematics, Beijing 100088, China.; ^8^ HEDPS, CAPT, College of Engineering, Peking University, Beijing 100871, China.

## Abstract

The advent of machine learning (ML) in computational chemistry heralds a transformative approach to one of the quintessential challenges in computer-aided drug design (CADD): the accurate and cost-effective calculation of atomic interactions. By leveraging a neural network (NN) potential, we address this balance and push the boundaries of the NN potential’s representational capacity. Our work details the development of a robust general-purpose NN potential, architected on the framework of DPA-2, a deep learning potential with attention, which demonstrates remarkable fidelity in replicating the interatomic potential energy surface for drug-like molecules comprising 8 critical chemical elements: H, C, N, O, F, S, Cl, and P. We employed state-of-the-art molecular dynamic (MD) techniques, including temperature acceleration and enhanced sampling, to construct a comprehensive dataset to ensure exhaustive coverage of relevant configurational spaces. Our rigorous testing protocols, including torsion scanning, structure relaxation, and high-temperature MD simulations across various organic molecules, have culminated in an NN model that achieves chemical precision commensurate with the highly regarded density functional theory model while substantially outstripping the accuracy of prevalent semi-empirical methods. This study presents a leap forward in the predictive modeling of molecular interactions, offering extensive applications in drug development and beyond.

## Introduction

In computer-aided drug design (CADD), the precise computation of intra-molecular interactions is not merely a detail—it is a cornerstone that underpins the fidelity of molecular modeling. This critical aspect determines the success of virtual screening and the identification of promising lead compounds, accelerating the journey from conceptual frameworks to clinical applications. However, this task is challenged in balancing the accuracy and computational cost of the methods one uses [1]. Existing methods generally can be divided into 2 types; one of them is the fully quantum mechanical (QM) method, such as density functional theory (DFT) [2,3] or post-Hartree-Fock [4] methods, which can accurately describe the intricate quantum properties of electrons, but their computational cost prohibits their application to large systems on a long time scales, despite the increasing computational power in recent years. Another type is the classical force field method, which has been widely used for macromolecules or large databases of molecules, but at the compromise of computational accuracy [5]. Therefore, developing an efficient method that can provide QM accuracy at a limited computational cost is of great significance.

Machine learning (ML) methods have been successfully used in various chemistry, biology, and physics research applications. In particular, the strategy pioneered by Behler and Parrinello (BP) [6], which made use of a deep neural network (NN) to represent the interatomic potential energy surface (PES) of the systems, introduces a promising methodology for refining the accuracy of molecular interaction calculations. In this method, the parameters of the NN were trained on a given dataset, which typically includes a vast number of molecular configurations along with their corresponding properties, such as energies and forces, calculated using quantum chemical methods. Therefore, the resulting NN can offer accuracy comparable to that of quantum chemical calculations, but with an efficiency improved by 3 to 4 orders of magnitude.

Since Behler and Parrinello’s work, different NN potentials have been developed to predict the energies and forces for drug-like molecules [7–16]. For instance, the ANI-1 model [7] that shares a similar design as the BP’s model was introduced in 2017 by Smith, Isayev, and Roitberg. By improving and expanding the ANI-1x model, some new potentials including ANI-1x [8], ANI-1cc [9], ANI-2x [10], and Schrödinger-ANI [17] were constructed. The ANI-2x potential can handle molecules containing 7 elements: H, C, N, O, F, Cl, and S. This extends the capability compared to ANI-1, ANI-1x, and ANI-1cc potentials, by including 3 additional elements: S, F, and Cl. Relative to ANI-2x, the Schrödinger-ANI potential additionally covers phosphorus. Recent advancements in NN models, such as AIMNet2 [16], MACE-OFF23 [18], and Nutmeg [19], have substantially enhanced the accuracy of NN models in pharmaceutical molecular simulation tasks.

Despite substantial advancements in the development of ML potential models for drug-like molecules, creating a universally accurate ML model that encompasses the vast chemical and conformational space of these molecules remains a considerable challenge. The potential number of drug-like molecules is estimated to be around 10^60^ [20], and this does not even take into account the configuration ensemble for each molecule. Achieving uniform accuracy across the chemical and configurational spaces necessitates exceptional representational capabilities for the ML model. Furthermore, the selection of a large number of atomic configurations in the training set often considerably slows down the training process. For example, for the original ANI-1 model, 22 million configurations are required in the training set to reproduce the energies and forces with ωB97X/6-31G* accuracy. Even with the assistance of an active learning strategy, 5.5 million configurations are still needed (dataset for the ANI-1X model). Identifying methods to reduce the number of configurations in the training set while simultaneously maintaining or even expanding the represented chemical and conformation space of drug-like molecules remains a nontrivial task.

In this study, we developed an ML potential model, named DPA-2-Drug, designed to accurately predict atomic interactions in drug-like molecules containing 8 elements: H, C, N, O, S, F, Cl, and P. We employed the DPA-2 architecture [21], known for its exceptional representational abilities, to model the PES. We utilized a concurrent learning approach combined with temperature acceleration and enhanced sampling techniques to generate a compact training dataset without sacrificing accuracy. The resulting DPA-2-Drug model was benchmarked on a variety of organic molecules for different applications, including torsion scanning, structure relaxation, and high-temperature molecular dynamics (MD) simulations. Our results demonstrate that the DPA-2-Drug model achieves chemical accuracy compared to our reference DFT calculations at the level of ωB97XD/6-31G**, despite using only approximately 1.4 million conformations of 0.1 million molecules in our training set.

## Results and Discussion

**Fig. 1.**
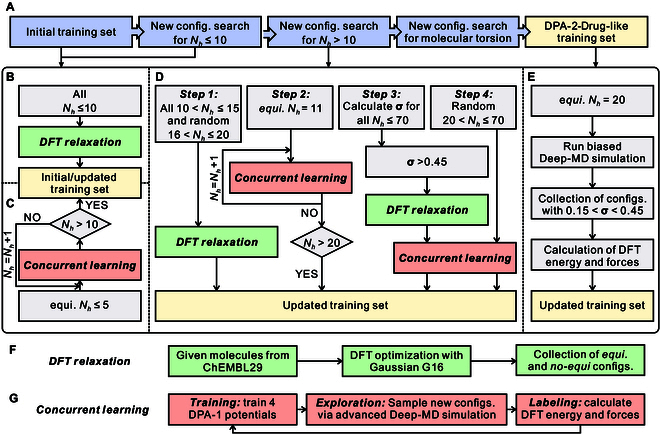
The flowchart for automatic collection of the drug-like training set. (A) Overall workflow. (B) Initial training data. (C) Workflow for sampling molecules with 10 or fewer heavy atoms. (D) Workflow for sampling molecules with more than 10 heavy atoms. (E) Sampling of molecular torsion. (F) DFT relaxation process. (G) Concurrent learning workflow.

The DPA-2-Drug model was trained using a dataset, detailed in Table 1, constructed via a concurrent learning algorithm, as illustrated in Fig. 1 and further explained in the Methods section. To evaluate the accuracy of the DPA-2-Drug compared to DFT reference calculations, we have performed extensive benchmarks on a range of applications, including torsion scanning, structure relaxation, and high-temperature MD simulations. For the torsion profile, we benchmarked the DPA-2-Drug potential on 3 typical torsion datasets of Genentech [22], Biaryl [23] drug fragments, and TorsionNet-500 [24]. To illustrate the transferability and robustness of the DPA-2 potential, we considered molecules in the test set of structure relaxation and MD simulations containing 20 to 70 heavy atoms, which are much larger than most of the molecules contained in the training set. For MD simulations, we have tested a much wider configurational space than those encountered in practical applications by running simulations with a high temperature of 700 K. For comparison, we also show the performance of the semi-empirical methods (such as PM6 and GFN2-xTB) in the benchmarks of structure relaxation and high-temperature MD simulations.

**Table 1.** Detailed information for the training set

	Chemical space	Configurational space		Total ^a^
	ChEMBL29	DFT opt.	MD sim.	
$N_{h}\leq 10$ ^b^	8,878	30,092		30,092
$N_{h}\leq 10$	-	-	186,450	186,450
$10<N_{h}\leq 20$	81,835	365,359	337,903	703,262
$10<N_{h}\leq 70$ ^c^	~6,500	41,975	89,781	131,756
$20<N_{h}\leq 70$	-	-	114,614	114,614
$N_{h}=20$ ^d^		37,085	160,328	197,413
Total	97,213	474,511	889,076	1,363,587

^a^ Total number of atomic configurations in the training set.

^b^ Initial training set.

^c^ Selected via model deviation calculation.

^d^ Molecular torsion.

### Torsion profiles

Having an accurate method to calculate the PES for molecular torsion is critical for screening ligands that could bind specifically to a target protein. To evaluate the performance of the DPA-2-Drug potential model in predicting torsion profiles, we conducted a validation using 3 widely recognized molecular torsion datasets: Genentech [22], Biaryl drug fragments [23], and TorsionNet-500 dataset [24]. These datasets are representative torsions typically found in small drug-like molecules and including a wide range of pharmaceutically relevant chemical space. For the Genentech torsional dataset, it consists of $N_{\text{mols}}=62$ molecules containing 7 elements of H, C, N, O, F, Cl, and S. Each molecule has $N_{\text{bins}}=36$ conformations generated by rotating one of the single bonds in 10° increments. The Biaryl torsional dataset tested here is the same as those used by Lahey et al. [25], in which $N_{\text{mols}}=88$ molecules containing 5 elements of H, C, N, O, and S are included. Each molecule has $N_{\text{bins}}=72$ conformations generated by rotating the single bond connecting 2 rings in 5° increments. The TorsionNet-500 dataset contains $N_{\text{mols}}=500$ different molecules with elements H, C, N, O, F, S, and Cl. Each molecule has $N_{\text{bins}}=24$ conformations generated by rotating one of the single bond in 15° increments.

In this study, we obtained torsional PESs for the Genentech and Biaryl datasets by performing constrained geometry optimizations at specific torsion angles. For the Genentech dataset, we used the published optimized structures at various torsion angles along each PES as the starting points for our optimization process. For the Biaryl dataset, we initiated a torsion scan from the published equilibrium geometries, where the structure optimized at each torsion angle served as the initial configuration for the subsequent conformation. For instance, the optimization of the 5° conformation started with the optimized configuration of the 0° conformation. This approach was consistently applied to all conformations from 0° to 360°. For the TorsionNet-500 dataset, we employed conformations from the original dataset [24], which were optimized under constrain using the B3LYP/6-31G(d) level of DFT. The accuracy of all NN methods was then evaluated and compared using this consistent set of conformations. It is important to note that the test configurations were generated by relaxing structures with constrained torsion angles, resulting in configurations that are largely absent from the training dataset, which was developed through structure relaxation and MD sampling. While there is a minimal risk of data leakage—specifically if a constrained torsion angle coincides with that of a relaxed structure included in the training set—such occurrences are unlikely to substantially affect our conclusions. This is because the errors are assessed across all sampling points in the torsion angle scan, and the saddle configurations are definitively excluded from the training datasets, as they are not local minima of the PES.

**Fig. 2.**
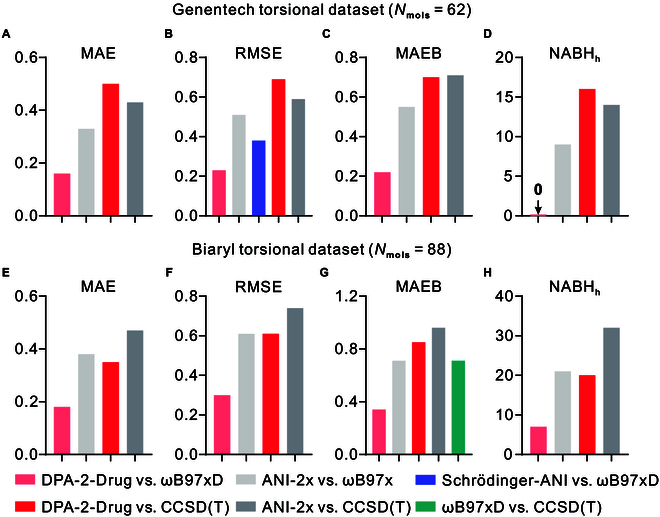
Comparison of the mean absolute error (MAE; A and E) of energy, root mean square error (RMSE; B and F) of energy, and MAE of the torsional barrier height (MAEB; C and G) between the DPA-2-Drug (this work) and the reference DFT methods on the torsional dataset of Genentech [10,22] and Biaryl drug fragments [25]. NABH*_h_* (D and H) refers to the number of torsional PESs for which the model prediction of potential barrier height has an error of more than 1 kcal/mol. The DFT method ωB97xD used in DPA-2 and Schrödinger-ANI models corresponds to ωB97xD/6-31G** and ωB97xD/6-31G*, repectively. The ωB97x method used in the ANI-2x model refers to ωB97x/6-31G*. For the calculation of the Genentech torsional dataset and the Biaryl torsional dataset, the CCSD(T) methods used are CCSD-(T)*/CBS//MP2/6-311+G** and CCSD-(T)*/CBS//RIMP2/def2-TZVP, respectively.

**Fig. 3.**
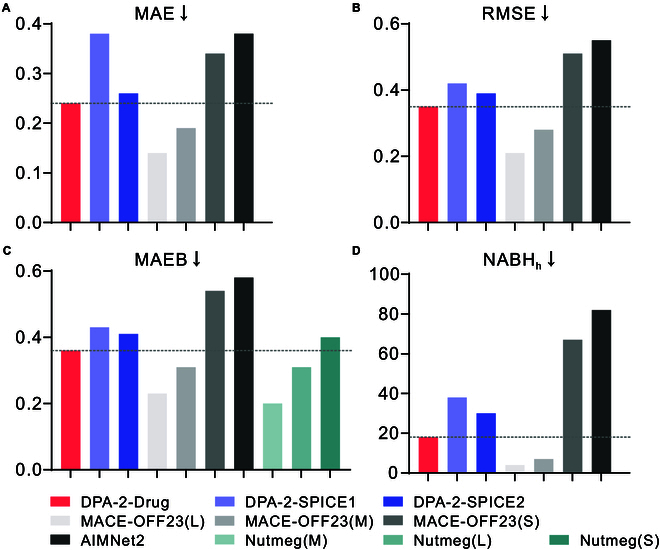
Comparison of (A) MAE, (B) RMSE, and (C) MAEB errors between NN models [DPA-2-Drug, MACE-OFF23(L/M/S), DPA-2-SPICE1/DPA-2-SPICE2 [18], AIMNet2 [46], and Nutmeg(L/M/S) [19]] and its reference DFT methods on the torsional dataset of TorsionNet-500. (D) NABH*_h_* refers to the number of torsional PESs (total: $N_{\text{mols}}=500$) for which the model prediction of potential barrier height has an error of more than 1 kcal/mol. The reference DFT methods for the DPA-2-Drug and AIMNet2 models are ωB97xD/6-31G** and ωB97M-D3/def2-TZVPP, respectively. All other models use ωB97M-D3(BJ)/def2-TZVPPD as their reference DFT method. Train dataset details: DPA-2-Drug: the dataset reported in this work; DPA-2-SPICE1: a subset of the SPICE1 dataset with unreasonable structures removed; MACE-OFF23(L/M/S): as described in [45], this dataset includes the neutral subset of SPICE1 with problematic structures removed, along with larger structures (50 to 90 atoms) from the QMugs dataset, and water clusters containing up to 50 water molecules; DPA-2-SPICE2 and Nutmeg(L/M/S) models: the SPICE2 dataset; AIMNet2: the dataset described in [46].

Figure 2 compared the energy errors between the potential DPA-2-Drug, ANI-2x [10,25], and Schrödinger-ANI [17] potential against its own reference level of DFT theory (ωB97xD/6-31G** for DPA-2-Drug, ωB97x/6-31G* for ANI-2x, and ωB97xD/6-31G* for Schrödinger-ANI). Moreover, the errors between different ML potentials and available CCSD(T) [22,25] results were also provided. Figure 3 presents a comparative analysis of the energy prediction accuracy for the DPA-2-Drug, DPA-2-SPICE1/2, MACE-OFF23, AIMNet2, and Nutmeg models. The errors are computed relative to the reference level of DFT used for labeling the training data: ωB97xD/6-31G** for DPA-2-Drug, ωB97M-D3(BJ)/def2-TZVPPD for SPICE1/2 models, and ωB97M-D3/Def2-TZVPP for AIMNet2.

We established 4 error metrics to assess the overall performance. The first 2 metrics are mean absolute error (MAE) and root mean square error (RMSE) for a given torsion dataset. These metrics can be calculated as follows:

$$\mathrm{MAE}=\frac{1}{N_{\text{mols}}}\sum_{j}^{N_{\text{mols}}}\frac{1}{N_{\text{bins}}}\sum_{i}^{N_{\text{bins}}}\left|E_{\mathrm{ij},\mathrm{DFT}}^{r},-,E_{\mathrm{ij},\mathrm{ML}}^{r}\right|,$$ (1)

$$\text{RMSE}=\sqrt{\frac{1}{N_{\text{mols}}}\sum_{j}^{N_{\text{mols}}}\frac{1}{N_{\text{bins}}}\sum_{i}^{N_{\text{bins}}}{\left(E_{\mathrm{ij},\mathrm{DFT}}^{r}-E_{\mathrm{ij},\mathrm{ML}}^{r}\right)}^{2}},$$ (2)

where $E_{\mathrm{ij}}^{r}$ is the relative energy of the *i*th point on the PES of molecule *j*. For the Genentech torsion dataset, we defined it as the energy difference between the *i*th point and the first point on the PES. For the Biaryl drug fragment, we defined it as the energy difference between the *i*th point and the PES minimum energy point.

Our third metric is the MAE of the torsional barrier height (MAEB) for the ML potential relative to their reference DFT method.

$$\text{MAEB}=\frac{1}{N_{\text{mols}}}\sum_{j}^{N_{\text{mols}}}\left|\Delta E_{j,\mathrm{DFT}}^{‡}-\Delta E_{j,\mathrm{ML}}^{‡}\right|,$$ (3)

where $\Delta E_{j}^{‡}$ is the barrier height of the torsional rotation for molecule *j*, defined as the difference between the minimum and the maximum energy points on the PES.

In addition, we calculated the number of torsional PESs for which the absolute barrier height predicted using the ML potential has an error of more than chemical accuracy (1 kcal/mol) compared to the reference DFT method. Here, we named the metric as NABH*_h_*.

As observed in Fig. 2, for all metrics of both datasets, DPA-2-Drug versus ωB97xD outperforms ANI-2x versus ωB97x and Schrödinger-ANI versus ωB97xD. This suggests that the DPA-2-Drug model has superior fitting and generalization accuracy compared to ANI-2x and Schrödinger-ANI. Notably, the DPA-2-Drug versus ωB97xD mean absolute error in barrier height (MAEB) for the Genentech torsional dataset is 0.22 kcal/mol, and none of torsional PESs (among 62) has a barrier height error of more than 1 kcal/mol (NABH*_h_* = 0). This indicates that our DPA-2-Drug model can effectively reproduce molecular torsions with DFT-level accuracy. The comparison of torsional PES for each molecule in Genentech and Biaryl torsional datasets calculated with DPA-2-Drug and our reference DFT method is shown in Figs. S1 and S2, respectively. When compared to CCSD(T) results, the performance of DPA-2-Drug is comparable to that of ANI-2x on average. Although the error of DPA-2-Drug versus CCSD(T) is higher than DPA-2-Drug versus DFT, it still achieved chemical accuracy. One could substantially improve the accuracy of DPA-2-Drug by providing more accurate CCSD(T) labels and using techniques such as transfer leaning, similar to [9].

The DPA-2-Drug model was benchmarked against the MACE-OFF23(L/M/S), AIMNet2, and Nutmeg(L/M/S) models using the TorsionNet-500 benchmark. The MAE, RMSE, and MAEB errors and NABH*_h_* are compared and presented in Fig. 3. The trends across all metrics are consistent: The DPA-2-Drug model exhibits higher accuracy than the AIMNet2, MACE-OFF23(S), and Nutmeg(S) models but lower accuracy than the MACE-OFF23(M/L) and Nutmeg(M/L) models. Additionally, the DPA-2 model was trained on the SPICE1 and SPICE2 datasets using the same model architecture and training hyperparameters to investigate the effect of the training dataset on model accuracy. It was observed that the DPA-2-Drug model trained with the dataset proposed in this work outperforms the DPA-2 models trained on SPICE1 and SPICE2, indicating that the proposed dataset is more suitable for torsion profile predictions. Moreover, the DPA-2 model trained on SPICE2 showed higher accuracy than the one trained on SPICE1, suggesting that SPICE2 provides broader coverage of conformations than the SPICE1 dataset.

Furthermore, we evaluated the performance of the DPA-2-Drug model using 2D torsion profiles for 4 molecules containing H, C, N, O, F, Cl, and S, as described in [10]. For each molecule, 2 dihedral angles were predefined and rotated in 10° increments, yielding a total of 1,296 structures (36 × 36) per torsion profile. Each structure was initially optimized using DFT, followed by re-optimization with the DPA-2-Drug model while keeping the dihedrals fixed along the rotation path. The resulting 2D torsion profiles from both the DPA-2-Drug and DFT calculations, as well as the corresponding MAE and RMSE energy errors, are shown in Fig. S3 and Table S1. In all cases, the DPA-2-Drug model achieved energy MAE within the chemical accuracy threshold. The model’s performance was comparable to, or even superior to, ANI-2x [10], particularly for the cysteine dipeptide systems, which exhibited a broader energy range (up to 30 kcal/mol).

### Structure relaxation

A crucial application in ligand screening for drug discovery involves identifying the energetically more stable configuration, i.e., the local minima of the PES. To illustrate the utility of the DPA-2-Drug potential in this task, we performed relaxation calculations on a test dataset of 200 drug-like molecules randomly selected from the ChEMBL29 dataset. These molecules, containing between 20 and 70 heavy atoms, have distinct SMILES strings compared to those in the training dataset. We employed various methods for optimization, including DPA-2-Drug, GFN2-xTB, and our reference DFT method ωB97xD, and then compared the resemblance of these optimized geometries obtained by the different methods. In this instance, we did not evaluate the ANI-2x model, as its coverage of chemical elements is insufficient to encompass all the molecules in the test dataset. The initial 3-dimensional (3D) structures of these molecules were converted from SMILES strings and followed by preliminary MMFF94 [26] optimization using the RDkit software package [27]. For simplicity, we marked these molecules as RDkit-generated ones.

For relatively large molecules, the energy landscapes contain numerous local minima, and structure relaxation algorithms do not guarantee finding the global minimum. Consequently, the structure optimization procedure is highly sensitive to the choice of initial configurations. In this work, we introduce 3 distinct approaches for selecting initial configurations.

Case1: Directly relax the RDkit-generated structures by using the DPA-2-Drug model, the semi-empirical GFN2-xTB, and our reference DFT method ωB97xD, respectively.

Case 2: Starting from the GFN2-xTB optimized geometries in case 1, relax the geometries by using DPA-2-Drug model and our reference DFT method, respectively.

Case 3: Starting from the DFT relaxed geometries in case 2, the geometries are further relaxed by DPA-2-Drug and GFN2-xTB for comparison.

To quantify the discrepancy between optimized geometries, we calculated the root mean square deviation (RMSD), bond lengths, angles, and torsion MAEs of DPA-2-Drug or GFN2-xTB against DFT and presented the results in Table 2. In the table, DPA-2_s.f.RDkit_ and GFN2-xTB_s.f.RDkit_ denotes the difference between the DPA-2-Drug and DFT relaxed structures and that between the GFN2-xTB and DFT relaxed structures in case 1, respectively. The “s.f” is abbreviated for “starting from”. DPA-2_s.f.XTB_ and DPA-2_s.f.DFT_ denote the difference between DPA-2-Drug and DFT in cases 2 and 3, respectively.

**Table 2.** Comparison of optimized geometries obtained by DPA-2 model and GFN2-xTB with those obtained using reference DFT method of ωB97xD. DPA-2_s.f.RDkit_, DPA-2 _s.f.XTB_, and DPA-2 _s.f.DFT_ refer to DPA-2-Drug against DFT results obtained starting with RDkit-generated, GFN2-xTB-optimized, and DFT-optimized geometries, respectively. GFN2-xTB_s.f.RDkit_ and GFN2-xTB_s.f.DFT_ indicate GFN2-xTB against DFT results obtained starting with RDkit-generated and DFT-optimized molecule conformations, respectively.

Metric (MAE)	XTB_s.f.RDkit_	XTB _s.f.DFT_	DPA-2_s.f.RDkit_	DPA-2_s.f.XTB_	DPA-2_s.f.DFT_
RMSD (Å)	1.25	0.81	0.86	0.57	0.25
Bond length (Å)	0.00585	0.00572	0.00117	0.00100	0.00083
Angles (°)	0.68	0.61	0.30	0.25	0.20
Torsion (°)	12.53	8.39	7.66	5.59	3.20

In case 1, the DPA-2-Drug model has lower errors in RMSD, bond length, angle, and torsional MAEs than GFN2-xTB method (DPA-2_s.f.RDkit_ versus GFN2-xTB_s.f.RDkit_), which is one of the most often used methods for initial screening candidate drugs. Furthermore, our results demonstrate that the performance of DPA-2-Drug can be substantially enhanced if GFN2-xTB- and DFT-optimized geometries are used as starting points for relaxation, rather than RDkit-generated geometries. This is because the errors exhibit a clear trend: DPA-2_s.f.RDkit_ > DPA-2_s.f.XTB_ > DPA-2_s.f.DFT_. For the DPA-2_s.f.DFT_ case, the RMSD MAE between DPA-2-Drug and DFT-optimized geometries was only 0.23 Å, compared to 0.84 Å in the DPA-2_s.f.RDkit_ case. This result highlights the substantial impact of the quality of starting configurations on the resulting optimized geometries. In fact, our calculations reveal that when the initial configurations are far from the local minimum, relaxations using different methods are likely to converge to distinct local minima. The quality of the relaxed structures from DPA-2_s.f.DFT_ can be further substantiated by examining the energy differences between DPA-2-Drug and DFT-optimized geometries. The average absolute energy difference is reported to be 0.8 kcal/mol, as calculated using the DPA-2-Drug model, with 160 of 200 structures (80%) exhibiting an energy difference within 1 kcal/mol. Based on these findings, the DPA-2-Drug model is a useful tool for predicting high-quality conformations. This method enables an effective structure relaxation for drug development in the context of CADD.

### MD trajectory

Finite temperature MD simulations is one of the most important applications of the ML potentiil models in the CADD. To illustrate the utility and stability of our DPA-2-Drug model in MD simulations, here we calculated the MAE and RMSE of relative energy and force errors for configurations extracted from higher-temperature MD trajectories. The relative energies of specific configurations were calculated with respect to the first configuration on each trajectory. These simulations were performed in the canonical ensemble (NVT ensemble) with the production DPA-2-Drug model at temperatures of 750 K, which is much higher than those used in typical applications. The energy and forces of these configurations were then labeled using our reference DFT ωB97XD/6-31G** and some semi-empirical methods, including PM6 and GFN2-xTB. For comparison, we further labeled energy and forces using the ANI-2x model and its reference DFT of ωB97X/6-31G* for some configurations whose chemical elements are covered in ANI-2x model.

**Fig. 4.**
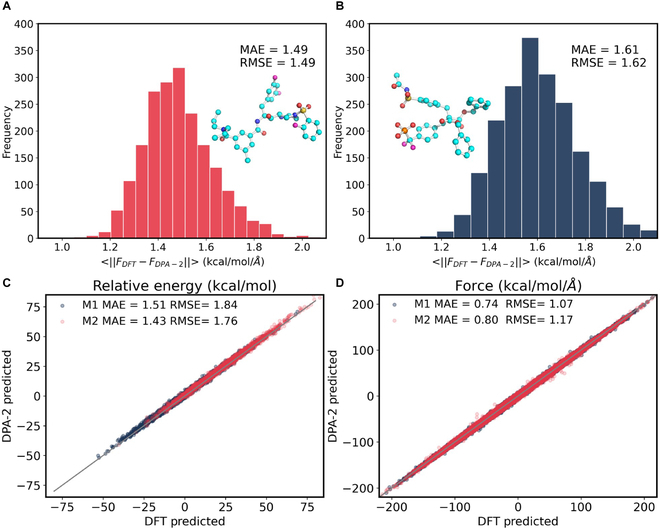
(A and B) Distribution of the magnitude of the error in force, represented as $\langle ∥F_{\mathrm{DFT}}-F_{\mathrm{DPA}2}∥\rangle$, for molecules M1 (A) and M2 (B) along the NVT trajectories at 750 K, respectively. (C and D) Comparative analysis of the relative energies (C) and forces (D) predicted by the DPA-2-Drug model versus those derived from the reference DFT calculations along the NVT trajectory, respectively. Two typical drug-like molecules M1 and M2 from the ChEMBL29 dataset (not included in the training set) have been chosen as examples and were given as ball-and-stick type. These 2 molecules contain all atomic elements (C, H, N, O, F, S, Cl, and P) considered in this work. We ran 3 ns for each trajectory, and 2,000 configurations were selected at an equal interval from the final 2 ns of the simulation. Relative energies were calculated referring to the first configuration on each trajectory. The magnitude of an error δF was calculated by $\langle \;,∥\;\delta F\;∥\rangle =\frac{1}{N}\sum_{i}^{N}\sqrt{\left(\delta F_{\mathrm{ix}}^{2}+\delta F_{\mathrm{iy}}^{2}+\delta F_{\mathrm{iz}}^{2}\right)}$ , where *N* is the number of atoms in each molecule.

Specifically, 2 different datasets, labeled by ChEMBL-MD-short and ChEMBL-MD-long, were prepared. The ChEMBL-MD-short was collected by running short-time simulations (25 ps) for 200 molecules randomly selected from the ChEMBL29 dataset. These molecules have a heavy atom number of $20<N_{h}\leq 70$ (not included in the training set, as they feature distinct SMILES strings compared to those in the training dataset) and contain all atomic elements (C, H, N, O, F, S, Cl, and P) being considered in this study. The number of atoms per molecule varies from 34 to 150 with an average value of 72.05. From each trajectory, we selected 50 configurations at equal intervals, resulting in a diverse dataset containing 10,000 configurations. Configurations in the ChEMBL-MD-long dataset were extracted from two 3-ns long-time trajectories performed on 2 molecules, named by M1 and M2, as shown in Fig. 4A and B, respectively. These 2 molecules were also from the ChEMBL29 dataset and were not included in the training dataset as well. In particular, M1 comprised 88 atoms containing 7 elements of C, H, N, O, F, S, and Cl, and M2 consisted of 81 atoms with 7 elements of C, H, N, O, F, S, and P. From each trajectory, we selected 2,000 configurations at an equal interval from the final 2 ns of the simulation for our benchmark dataset.

Three factors render this benchmark exceptionally challenging. One such factor is the high temperature of the simulations, which results in a considerably broader configurational space being sampled, including configurations far from equilibrium. Structures along the MD trajectories exhibit a wide range of relative energies, from −55 to 55 kcal/mol, indicating that these structures experience significant fluctuations. Another contributing factor is the diversity of simulation molecules, which possess a considerably larger number of atoms compared to those used in the training set. Moreover, some of these molecules contain benzene rings (or hybridized forms), making the accurate characterization of intramolecular π−π interactions essential. The success of this MD calculation depends on the ability of our DPA-2-Drug potential to accurately estimate the potential energies and atomic forces for these configurations.

Figure 4 gives the comparison of the relative force magnitude, energy, and force errors between the DPA-2 model and our reference DFT method of ωB97XD/6-31G** over configurations in ChEMBL-MD-long dataset. The MAE/RMSE of relative energy errors for molecules M1 and M2 is 1.51/1.84 and 1.43/1.76 in kcal/mol, respectively. The MAE/RMSE of force magnitude errors for molecules M1 and M2 are 1.49/1.49 and 1.61/1.62 in kcal/mol/Å, respectively. The MAE/RMSE of force component errors for molecules M1 and M2 is 0.74/1.07 and 0.80/1.17 in kcal/mol/Å, respectively. Importantly, we found that these errors did not increase as the simulation goes, even after long time scale simulations (3 ns). DPA-2-Drug was still sampling configurations that agree well with the reference DFT.

**Fig. 5.**
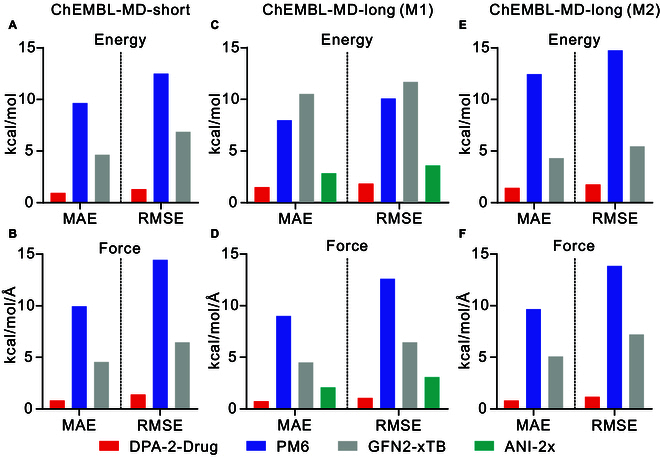
Comparison of MAE and RMSE between DPA-2-Drug, PM6, and GFN2-xTB against our reference DFT method of ωB97XD/6-31G** for both datasets of ChEMBL-MD-short and ChEMBL-MD-long, and between ANI-2x against its reference DFT method of ωB97X/6-31G* for molecule M1 in dataset of ChEMBL-MD-long. The molecule M2 is out of the chemical space of ANI-2x; thus, the accuracy by ANI-2x is not reported. Plots (A), (C), and (E) display the energy MAE and RMSE for the ChEMBL-MD-short, ChEMBL-MD-long (M1), and ChEMBL-MD-long (M2) cases, respectively. Meanwhile, plots (B), (D), and (F) show the force MAE and RMSE for the ChEMBL-MD-short, ChEMBL-MD-long (M1), and ChEMBL-MD-long (M2) cases, respectively.

The comparison of MAE and RMSE between DPA-2-Drug, PM6, and GFN2-xTB against our reference DFT method of ωB97XD/6-31G** for both ChEMBL-MD-short and ChEMBL-MD-long datasets, and between ANI-2x against its reference DFT method of ωB97X/6-31G* for molecule M1 are shown in Fig. 5. For both datasets, the DPA-2-Drug method has much lower MAE/RMSE errors compared to DFT for both relative energy and forces than both PM6 and GFN2-xTB. In our studies, the performance of GFN2-xTB is slightly better than that of PM6 on average. For molecule M1, DPA-2-Drug outperforms ANI-2x, especially in terms of force MAE/RMSE errors. When focusing on the DPA-2-Drug results, we found that the DPA-2-Drug MAE/RMSE of relative energy errors for dataset ChEMBL-MD-short is smaller than that for ChEMBL-MD-long. This is expected since the average number of atoms per molecule for dataset ChEMBL-MD-long is bigger than that for the ChEMBL-MD-short dataset and the quantity of MAE energy error will increase as the number of atoms per molecule grows. For the DPA-2-Drug MAE/RMSE forces, these 2 datasets have comparable performance, for example, the DPA-2-Drug MAE force errors for datasets ChEMBL-MD-short and ChEMBL-MD-long were both around 0.7 kcal/mol/Å. The good performance of DPA-2-Drug on the 2 datasets representing diversity and stability suggests that DPA-2-Drug is a stable and powerful potential that can reproduce the PES of drug-like molecules well at finite temperatures with reference DFT accuracy.

Table 3 presents a comparison of MD simulation speeds for the DPA-2-Drug, MACE-OFF23, and AIMNet2 ML models, alongside the semi-empirical method GFN2-xTB. These measurements were derived from 3 independent 5,000-step NVT MD simulation conducted on an NVIDIA A800 GPU for the ML models and on an Intel Xeon Gold 6240R CPU for GFN2-xTB. All simulations were executed using the atomic simulation environment [28]. For the DPA-2-Drug model, the MD simulation speed was 52 ms per step for a molecule with 50 atoms and 51 ms per step for a molecule with 148 atoms. The simulation speed did not substantially change with system size, likely due to the relatively small number of atoms, which does not fully utilize the GPU’s computational capacity. A noticeable performance decrease to 63 ms per step was observed only when the system size increased to 745 atoms. A similar phenomenon was observed for the MACE-OFF23 and AIMNet2 models. Among all the ML models, AIMNet2 demonstrated the fastest simulation speeds. The DPA-2-Drug model was slower than the MACE-OFF23 models for relatively small systems but showed an advantage over all the MACE-OFF23 models for relatively large systems. The performance of the semi-empirical GFN2-xTB method, tested on CPUs, saturated at 8 CPU cores and was inferior to all the ML models for systems with more than 50 atoms.

**Table 3.** Comparison of molecular dynamics simulation speeds for various NN models, DPA-2-Drug, MACE-OFF23 (small, medium, and large configurations), and AIMNet2, across molecules with atom counts ranging from 50 to 1,490. The semi-empirical method GFN2-xTB, tested using different numbers of CPU cores, is included for comparison. All simulations were conducted using the atomic simulation environment [28]. Computation times per step (in milliseconds) represent averages from 3 independent trajectories, each comprising 5,000 time steps.

# Atoms	Time per MD step (ms) ↓						
	DPA-2-Drug ^a^	MACE-OFF23 ^a^			AIMNet2 ^a^	GFN2-xTB ^b^	
		Small (S)	Medium (M)	Large (L)		4 cores	8 cores
50	51.9	29.1	33.9	42.7	18.9	54.4	50.1
60	49.9	28.7	35.1	46.4	19.7	80.6	71.1
100	50.0	29.0	34.8	58.9	21.5	187.4	158.9
148	51.2	30.6	38.9	83.9	19.4	403.2	324.7
375	53.4	79.1	105.3	233.8	23.9	-	-
745	62.8	107.4	163.0	409.0	24.9	-	-
1,490	104.6	251.8	367.7	926.9	59.9	-	-

^a^ Simulations were run on an NVIDIA A800 GPU.

^b^ Simulations were run on an Intel Xeon Gold 6240R CPU.

## Conclusion

In this work, we introduced a deep learning potential, termed DPA-2-Drug potential, to reproduce the interatomic interactions for drug-like molecules containing 8 chemical elements (H, C, N, O, F, S, Cl, and P) with a DFT accuracy of ωB97XD/6-31G**. This potential was trained using our newly developed architecture of DPA-2-Drug on an elaborated training set. The chemical space of this training set was selected from the ChEMBL dataset containing 1 to 70 heavy atoms as summarized in Table 1, and the conformational space was automatically built using a successive concurrent learning technique as implemented in the open-source software platform Deep Potential GENerator (DP-GEN) [29]. Because of a combination of efficient model architecture and advanced MD including temperature acceleration and enhanced sampling simulation, a vast configuration space away from equilibrium was also included, especially along the torsion profile. We have shown that a total of approximately 0.1 million molecules of 1.4 million conformations was sufficient to represent the relevant molecular and conformational space needed to train the PESs of great interest for drug development in CADD.

We have advanced the DPA-2-Drug model through several applications crucial to drug development, such as torsion scanning, structure relaxation, and high-temperature MD simulations at 750 K. The model has demonstrated excellent accuracy across various organic molecules and outperforms the most widely used semi-empirical methods like GFN2-xTB and PM6. Notably, in applications like structure relaxation and MD simulations, the model adeptly handles molecules in the test set with 20 to 70 heavy atoms—substantially more complex than those in the training set.

The DPA-2-Drug model emerges as a promising tool in CADD, enabling precise predictions of atomic interactions. Despite its success, the model has limitations, such as its inability to process charged systems and radicals [30]. However, we are committed to refining the model to incorporate long-range electrostatic interactions and to expand its capabilities to simulate PESs for drug molecules within real protein and solution environments. This ongoing development underscores the model’s pivotal role in enhancing the accuracy and applicability of drug discovery.

## Methods

### Concurrent learning algorithm

In this work, we used the concurrent learning approach as implemented in the open-source software platform DP-GEN [29] to sample related configurations for our target drug-like training set. This algorithm has been demonstrated as a powerful tool for constructing ML potentials with a minimal number of reference data in a wide range of applications including chemistry [31–33], physics [34,35], and materials science [36–38]. As shown in Fig. 1G, each iteration of the concurrent learning procedure consists of the following 3 steps. We refer the readers to [29] for further details.

Step 1: Training. We trained an ensemble of ML potential models with distinct weights based on the updated training set of the latest iteration. Specifically, we trained 4 models using the DeePMD-kit package with the attention-based deep potential scheme [39], termed as DPA-1, which is able to give an accurate reproduction and prediction of DFT energies and atomic forces, especially for multi-component systems. To achieve relatively high-accuracy models with fewer training steps, we employed a training strategy wherein the parameters of the models from the previous iteration were utilized to initialize the models in the subsequent iteration. For the very first iteration, the model weights were randomly initialized using different seeds to ensure diversity in the ensemble. Please check the Supplementary Materials for more details about the training parameters.

Step 2: Exploration. We explored new relevant configurations via advanced MD simulation, including temperature acceleration and enhanced sampling techniques, to boost the sampling of molecular conformations. In these simulations, the interatomic potentials were calculated using one of the DPA-1 potentials obtained in step 1. Simulation temperatures and lengths were increased from 300 K to 750 K, and 1.5 ps to 20 ps, respectively, as the iteration progressed. Simulating at high temperatures up to 750 K is crucial to accelerate the sampling of more out-of-equilibrium configurations.

To estimate the reliability of potentials on configurations explored by these simulations, we measured the error indicator σ, which is the maximal standard deviation of the atomic forces predicted by the DPA ensemble. Being more precise, σ is defined by

$$\sigma =\max_{i}\sqrt{\frac{1}{N_{m}}\sum_{\alpha =1}^{N_{m}}∥F_{i}^{\alpha }-\bar{F_{i}}{∥}^{2}}$$ (4)

where $N_{m}=4$ denotes the number of the the models, $F_{i}^{\alpha }$ is the atomic force on the atom *i* predicted by the model α, and $\bar{F_{i}}$ is the average force on the atom *i* over the $N_{m}$ models. Hereafter, we call this error indicator σ model deviation.

To minimize the number of new relevant atomic configurations while ensuring maximum diversity for the training set, we used an adaptive strategy to select configurations for the training set. Specifically, 2 parameters were set. One is an upper bound ($\sigma_{u}$) of the model deviation. The configurations with a model deviation larger than $\sigma_{u}$ is usually associated with nonphysical configurations in which atoms are too close or correspond to improbable chemistry. The other parameter is the ratio of configurations to be treated as candidates ($r_{\text{candi}}$). In each iteration, the configurations with model deviation $\sigma >\sigma_{u}$ are firstly discarded, and then a $r_{\text{candi}}$ portion with the largest model deviation in the remaining configurations are treated as candidates. With this setup, a lower bound ($\sigma_{l}$) was obtained as the lowest model deviation value in the candidate set. In each iteration, 2,000 to 5,000 configurations were randomly subsampled from the candidate list and were added to the training dataset after being labeled by the next labeling step. In this work, we set $\sigma_{l}=0.5$ and adjusted the value of $r_{\text{candi}}$ between 0.10 and 0.15. 

Step 3: Labeling. We labeled the selected configurations in step 2 by calculating their DFT energies and atomic forces and then added them to the training set for the next iteration. Here, all DFT energies and atomic forces were calculated with Gaussian 16 software [40], using the hybrid meta-GGA (generalized gradient approximation) exchange-correlation functional of *w*B97XD [41] and 6-31G_**_ [42] basis set.

The criteria for stopping activation learning iterations are as follows. In each iteration, we monitored the evolution of the lower bound $\sigma_{l}$ value. An almost unchanged value in successive iterations implies that there are limited spaces for improving the quality of the training set. Once the fluctuation of this value is less than 0.01 over 8 iterations, we exit the loop. This ensures proper exploration of the configuration space.

### Construction of the training dataset

We chose the ChEMBL dataset, an open large-scale library of bioactive molecules with drug-like properties, as a starting point to build our target drug-like training set. Specifically, we used a filtered ChEMBL29 dataset [43] that excludes charged molecules, molecules with molecular weights greater than 1,000, or molecules containing elements other than the 8 elements H, C, N, O, F, Cl, S, and P. Molecules composed entirely of these 8 elements account for approximately ~94% of the total, amounting to around 1.88 million molecules. Hereafter, the ChEMBL29 dataset we refer to is the filtered one. Molecules in this dataset are supplied in the form of SMILES strings, we first converted them to 3D structures and then primarily optimized them with Merck molecular force field (MMFF94) [26] using the RDKit software package [27]. Based on this ChEMBL29 dataset, we used the concurrent learning algorithm to automatically sample the chemical and conformational spaces for the drug-like training set. The detailed flowchart is given in Fig. 1.

#### Initial training dataset

The collection of the drug-like training set started with the initial training set, which was obtained by first performing DFT relaxation following the process given in Fig. 1F on molecules with a heavy atom number, $N_{h}\leq 10$. Then, we collected 8,878 optimized structures and selected 21,214 nonequilibrium configurations generated during the DFT relaxation process, as shown in Fig. 1B. The whole process for expanding and refining the drug-like dataset can be summarized into the following 3 stages.

#### Molecules with number of heavy atoms *N_h_* ≤ 10

The initial training dataset consists solely of training data from relaxation paths. At this stage, we enriched the dataset using a concurrent learning algorithm to sample conformations within a temperature range of 300 to 750 K. We started by exploring the conformational space of molecules with a number of heavy atoms ($N_{h}$) less than or equal to 5. Subsequently, we incrementally increased $N_{h}$ by 1 and repeated the process until we explored the conformational space of molecules with 10 heavy atoms ($N_{h}=10$). The flowchart of these calculations is detailed in Fig. 1C. In the end, a total number of 186,450 structures were sampled via this workflow.

#### Molecules with number of heavy atoms *N_h_* ≤ 10

In this stage, we first gathered 365,359 training data points from the DFT relaxation paths of all ChEMBL29 molecules with heavy atoms in the range of $10<N_{h}\leq 15$ (totaling 58,784 molecules) and randomly selected 23,051 molecules with heavy atoms in the range of $16<N_{h}\leq 20$ (as shown in step 1 of Fig. 1D). We then performed concurrent learning calculations to sample the conformational space for these molecules. We started exploring the conformations of molecules with a heavy atom count of $N_{h}=11$. As the concurrent learning iteration converged, the number of heavy atoms ($N_{h}$) was increased by 1 until $N_{h}=20$ was reached, as illustrated in step 2 of Fig. 1D. In this way, we generated 337,903 conformations for the training set.

At this point, we calculated model deviation σ for all molecules with heavy atoms N≤70 from the ChEMBL29 dataset. This calculation was performed directly on MMFF94 [26] optimized 3D structures generated by the RDkit software [27].

We considered the chemical space of molecules with a model deviation value σ>0.45 (in total ~6,500) as they were not well represented by the training set. We then performed DFT relaxation and concurrent learning calculations to sample their conformation space (step 3 of Fig. 1D). In this way, we generated 131,756 additional data for the training set.

Furthermore, we also performed the concurrent learning for molecules randomly sampled from ChEMBL29 with heavy atoms $20<N_{h}\leq 70$ to enrich the chemical and conformational spaces of our training set. Here, in each iteration, the starting 3D structure for MD exploration was randomly chosen from the ChEMBL29 dataset and generated by the RDkit, rather than the equilibrium configurations optimized using the DFT method (step 4 of Fig. 1D). As a result, a total of 114,614 new structures were sampled here.

#### Molecular torsion

To improve the sampling of molecular torsions, we enlarged the training set by performing biased simulations using the latest DPA-1 model for 1,473 equilibrium molecules with heavy atoms $N_{h}=20$ randomly sampled from the ChEMBL29 dataset, as illustrated in Fig. 1E. These simulations were implemented by adding an external bias to the interatomic potential with the assistance of the on-the-fly probability enhanced sampling (OPES) [44]. In general, the bias potential is applied along a small set of collective variables (CVs), which are functions of the atomic coordinates $s=s\left(R\right)$ and should be wisely chosen to encode the relevant slow modes of the process for a successful simulation. In our case, to enhance sampling dihedral torsion, we delicately selected all single bonds made of atoms with more than one coordination number in each molecule as our CV, and in total, 6,117 CVs were generated. For each CV, we ran one independent simulation at 750 K for 100 ps by adding a bias potential of 70 kJ/mol.

From these biased trajectories, we then screened out 160,328 configurations with model deviation values between 0.15 and 0.45. Subsequently, DFT energy and forces calculations were performed. These simulations are critical for sampling the configuration space of dihedral torsion, given that the torsion of some dihedral torsion has high energy barriers and cannot be sampled on a limited time scale by simply increasing the simulation temperature. Furthermore, we also performed optimization calculations of the torsion scan with our reference DFT method for randomly selected 535 setups from the 6,117 biased calculation. For each setup, the torsional degree of freedom, i.e., the selected dihedral torsion, was fixed every 10°, and the remaining degrees of freedom were relaxed through the optimization process, resulting in 36 conformations. In the end, 37,085 configurations, including the equilibrium and nonequilibrium ones generated in the optimization progress, were added to the training set.

### Architecture of the DPA-2 model

In this study, we utilize the DPA-2 model architecture as described by Zhang et al. [21] to construct a production model. The DPA-2 model assumes that the system’s energy is the sum of atomic energy contributions, each derived from atomic representations of the atom’s chemical and configurational environment through a fitting deep NN. The model employs 2 types of atomic representations: single-atom and pair-atom. These representations are initialized based on the atom types and the relative positions of neighboring atoms within a cutoff radius via a repinit layer. They are subsequently evolved through multiple representation transformer (repformer) layers, which facilitate information exchange between neighboring atoms. Specifically, our work involves 6 stacked repformer layers to construct the DPA-2-Drug model. The repinit layer of the DPA-2-Drug model uses a large cutoff radius of 6 Å to gather information from distant neighbors, whereas the repformer layer employs a cutoff radius of 4 Å to enhance computational efficiency. Atomic forces are predicted by taking the negative gradient of the system energy with respect to atomic coordinates. The DPA-2-Drug model ensures smooth, conservative, and symmetry-preserving predictions. The DPA-2 architecture’s message-passing updates of representations are analogous to those in equivariant graph neural networks (GNNs) such as MACE-OFF23 [18] and Nutmeg [19]. The primary distinction lies in the use of symmetry operations and the gate attention mechanism for updating single-atom and pair-atom representations, respectively. For comprehensive details on the DPA-2 architecture, readers are recommended to consult the original paper by Zhang et al. [21].

### Training of the DPA-2-Drug model

Following the data construction workflow illustrated by Fig. 1, a drug-like training set comprising approximately 0.1 million molecules of 1.4 million atomic configurations was obtained, as summarized in Table 1.

The production DPA-2-Drug model was trained using this final training set, with detailed training information provided in Section S2.2. The code, training dataset, and the DPA-2-Drug model are publicly available. Please refer to Conclusion for more information. To examine the impact of different training datasets on model accuracy, we trained 2 additional DPA-2 models using the SPICE1 [45] and SPICE2 [19] datasets. Both models used the same architecture and hyperparameters as the DPA-2-Drug model.

## Data Availability

The DeePMD-kit package for utilizing the DPA-2-Drug model is open source and freely available at https://github.com/deepmodeling/deepmd-kit. The drug-like training set and our production DPA-2-Drug model are available on the AIS Square website https://www.aissquare.com/datasets/detail?pageType=datasetsname=Drug%28drug-like-molecule%29_DPA_v1_0&id=143 and https://www.aissquare.com/models/detail?pageType=models&name=DPA2-Drug-Model-v1&id=294, respectively.
