# Supplementary material for: Ab Initio Accuracy Neural Network Potential for Drug-Like Molecules
Source: Research (Wash D C). 2025 Aug 25;8:0837. doi: 10.34133/research.0837 (PMC12377527; doi:10.34133/research.0837)
Supplement: Supplementary 1 — Supplementary Text Fig. S1 to S3 Table S1 [file research.0837.f1.pdf]

# Supporting Information: Ab initio Accuracy Neural Network Potential for Drug-like Molecules

Manyi Yang,[†] Duo Zhang,[‡,¶] Xinyan Wang,[¶] Bowen Li,[§] Linfeng Zhang,[¶,‡] Weinan E,[‡,‖,⊥] Tong Zhu,[*,§] and Han Wang[*,#,@]

†*The Institute of Green Chemistry and Engineering, Nanjing University, Suzhou, Jiangsu 215163, China*

‡*AI for Science Institute, Beijing 100080, China*

¶*DP Technology, Beijing 100080, China*

§*Shanghai Engineering Research Center of Molecular Therapeutics & New Drug Development, School of Chemistry and Molecular Engineering, East China Normal University, Shanghai, 200062, China*

‖*Center for Machine Learning Research, Peking University, Beijing 100871, P.R. China*

⊥*School of Mathematical Sciences, Peking University, Beijing, 100871, P.R. China*

#*National Key Laboratory of Computational Physics, Institute of Applied Physics and Computational Mathematics, Beijing 100088, China*

@*HEDPS, CAPT, College of Engineering, Peking University, Beijing 100871, China*

E-mail: tongzhu.work@gmail.com; wang_han@iapcm.ac.cn

# Contents

**1 Computational Details** **S-3**

**2 Training Parameters** **S-3**

2.1 DPA-1 training . . . . . . . . . . . . . . . . . . . . . . . . . . . . . . . . . S-3

2.2 DPA-2 training . . . . . . . . . . . . . . . . . . . . . . . . . . . . . . . . . S-4

**3 2D Torsion Scan** **S-5**

**4 Profiles for Genentech torsional dataset** **S-6**

**5 Profiles for Biaryl Drug Fragments dataset** **S-7**

**6 2D Torsion Profiles** **S-8**

**References** **S-9**

# 1 Computational Details

All DFT-based calculations were performed using Gaussian 16 software.[S1] All DFT- and DPA-2-Drug-based relaxations were done using the Berny algorithm as implemented in Gaussian 16 software,[S1] in which GEDIIS[S2] was adopted for optimization. We used the DeepMD-kit package[S3,S4] for the training of the DPA-1 and DPA-2 —NN potentials. All DPA-based MD simulations were performed using the LAMMPS[S5] MD engine with the DeepMD-kit[S3,S4] software plugged in to describe the interatomic interactions. For enhanced sampling simulations, we used the PLUMED[S6] plugin patched with LAMMPS.[S5]

# 2 Training Parameters

## 2.1 DPA-1 training

The DPA-1 potentials were trained using the attention-based Deep Potential scheme.[S7] The cutoff radius was set to smoothly decay from 0.5 Å to 6.0 Å. The maximum possible number of neighbors in the cutoff was set to 20, and the number of layers in the attention scheme was set to 3. We used three hidden layers with [40, 80, 160] nodes/layer for the embedding network and three hidden layers with [240, 240, 240] nodes/layer for the fitting network, whereas the size of the embedding matrix was set to 16. The learning rate was set to decay from $1.0 \times 10^{-4}$ to $5.0 \times 10^{-8}$ and we used a batch size of 8. The prefactors of the energy and force terms in the loss function were set to change during the training from 0.25 to 2 and from 100 to 1, respectively, and the training steps were adjusted between $1.0 \times 10^{6}$ to $5.0 \times 10^{7}$. For the very first iteration, the starting learning rate was set to $1.0 \times 10^{-3}$, and the starting prefactors of the energy and force terms in the loss function were set to 0.02 and 1000.

## 2.2 DPA-2 training

The DPA-2-Drug model was trained with the DPA-2 framework,[S8] in which two primary components of *repinit* and *reformer* are included in the descriptor architecture. Specifically, In the *repinit* layer, the cutoff radius for two-body embeddings was smoothly decayed from 0.5 Å to 6.0 Å, while for three-body embeddings, it decayed from 3.5 Å to 4.0 Å. The maximum number of neighbors within the cutoff radius was set to 120 for two-body embeddings and 40 for three-body embeddings. The architecture comprised three hidden layers containing [25, 50, 100] nodes/layers for two-body embeddings and [2, 4, 8] nodes/layers for three-body embeddings. For the *repformer* layers, we used 6 layers, and the cutoff radius was set to decay from 3.5 Å to 4.0 Å smoothly. The maximum possible number of neighbors in the cutoff was set to 40. We designated the dimensions of the single-atom and pair-atom feature representations to be 128 and 32, respectively. For the gated multi-head self-attention layers, we opted for a dimension of 32 and configured both types of layers with four attention heads. The model utilized a residual-update mechanism, initializing the norm of the learnable vectors to 0.01. We used three hidden layers with [240, 240, 240] nodes/layers for fitting network.

The DPA-2-Drug model was subjected to three training iterations to enhance accuracy. In each iteration, an automated batch size of $\lceil \frac{256}{N} \rceil$ was employed, where $N$ represents the number of atoms in the system and $\lceil \cdot \rceil$ denotes the ceiling function, which rounds up to the nearest integer. The training process was conducted over $8.0 \times 10^6$ steps using four GPU cards. During the first iteration, the learning rate was set to decay from $1.0 \times 10^{-3}$ to $3 \times 10^{-8}$. The prefactors for the energy and force terms in the loss function were dynamically adjusted from 0.02 to 2 and from 1000 to 100, respectively. For the subsequent two iterations, the model was initialized with the parameters from the preceding iteration. The learning rate was then decayed from $1.0 \times 10^{-4}$ to $3 \times 10^{-8}$. Additionally, the prefactors for the energy and force terms in the loss function were modified from 2 to 100 and from 100 to 1, respectively, throughout the training process.

# 3 2D Torsion Scan

Table S1: Energy errors of mean-absolute-error (MAE) and root-mean-square-error(RMSE), between the DPA-2-Drug (this work) and the reference DFT methods. Errors are given in Kcal/mol. ANI-2x results from Ref.[S9] were also present here for comparison.

| | | ANI-2x[S9] | | DPA-2-Drug | |
|---|---|---|---|---|---|
| Structure | No.atoms | MAE | RMSE | MAE | RMSE |
| Cysteine dipeptide | 25 | 1.75 | 2.55 | 1.06 | 1.56 |
| DDT | 28 | 0.53 | 0.71 | 0.52 | 0.67 |
| Hexafluoroacetone | 10 | 0.09 | 0.11 | 0.24 | 0.42 |
| Bendamustine | 44 | 0.50 | 0.66 | 0.52 | 0.64 |

# 4 Profiles for Genentech torsional dataset

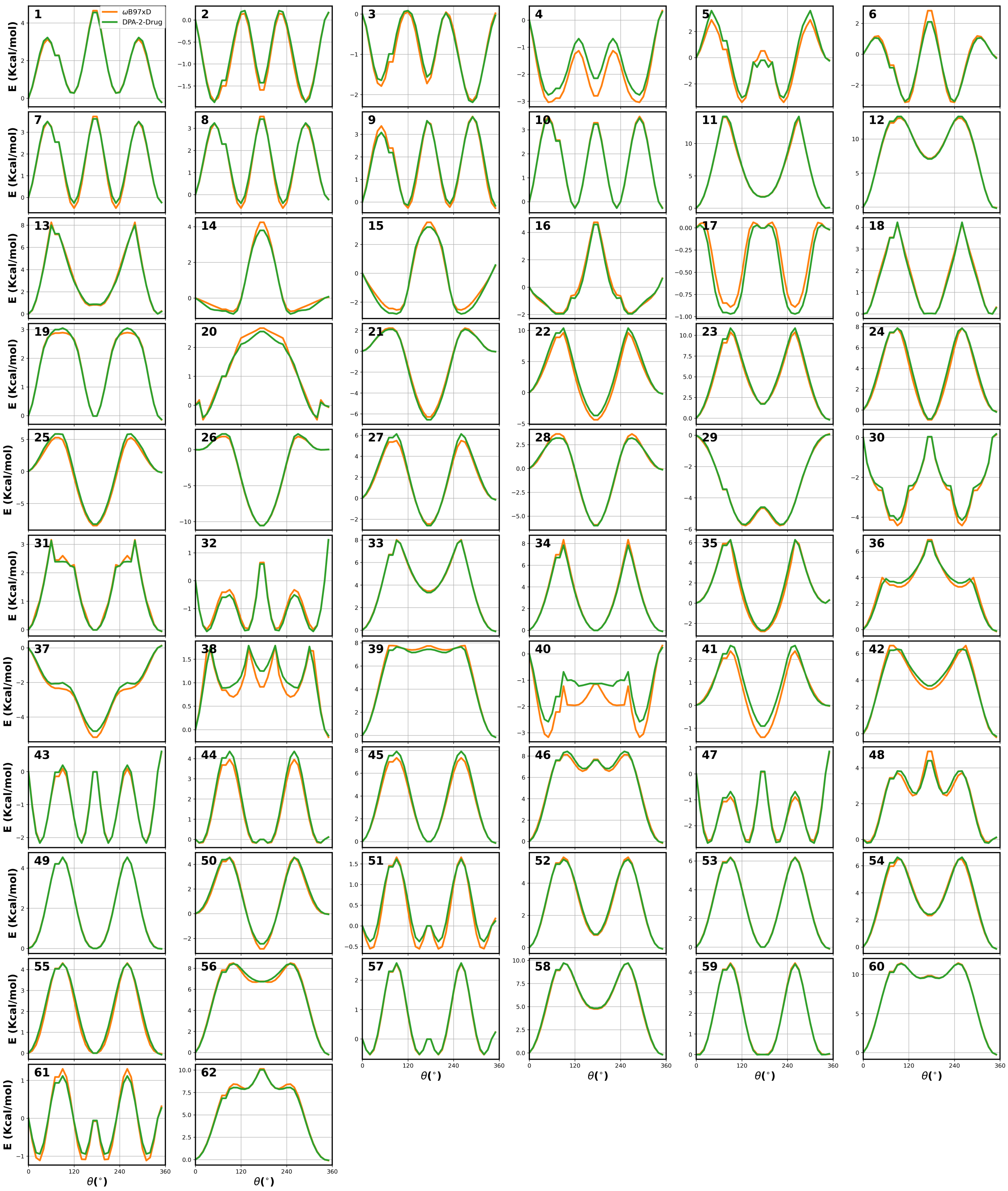


Figure S1: The comparison of torsional PES calculated with DPA-2-Drug potential (green) and the reference DFT method of $\omega$B97XD/6-31G** (orange) for each molecule in Genentech torsional dataset.

# 5 Profiles for Biaryl Drug Fragments dataset

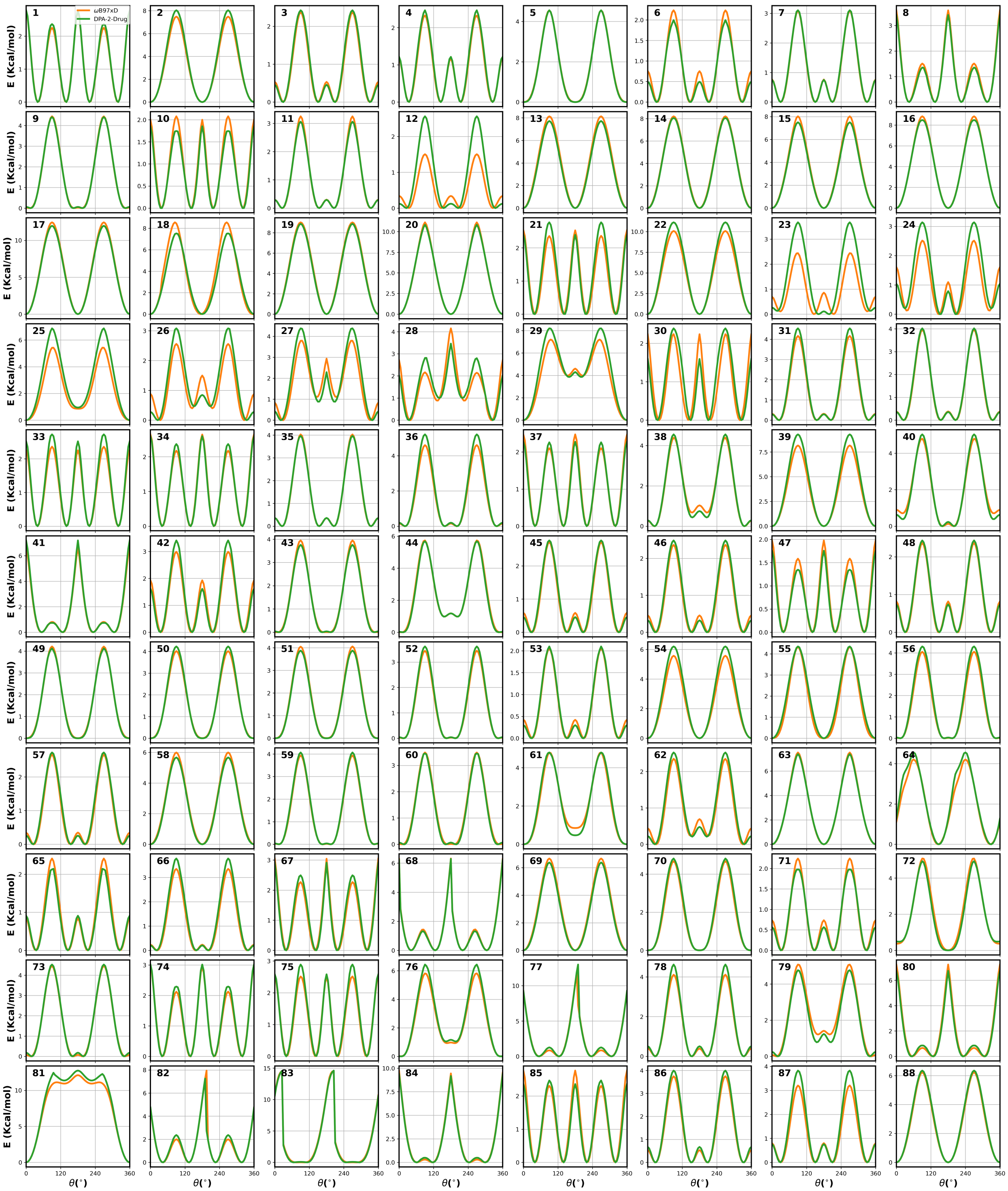


Figure S2: The comparison of torsional PES calculated with DPA-2-Drug potential (green) and the reference DFT method of ωB97XD/6-31G** (orange) for each molecule in Biaryl Drug Fragments dataset.

# 6 2D Torsion Profiles

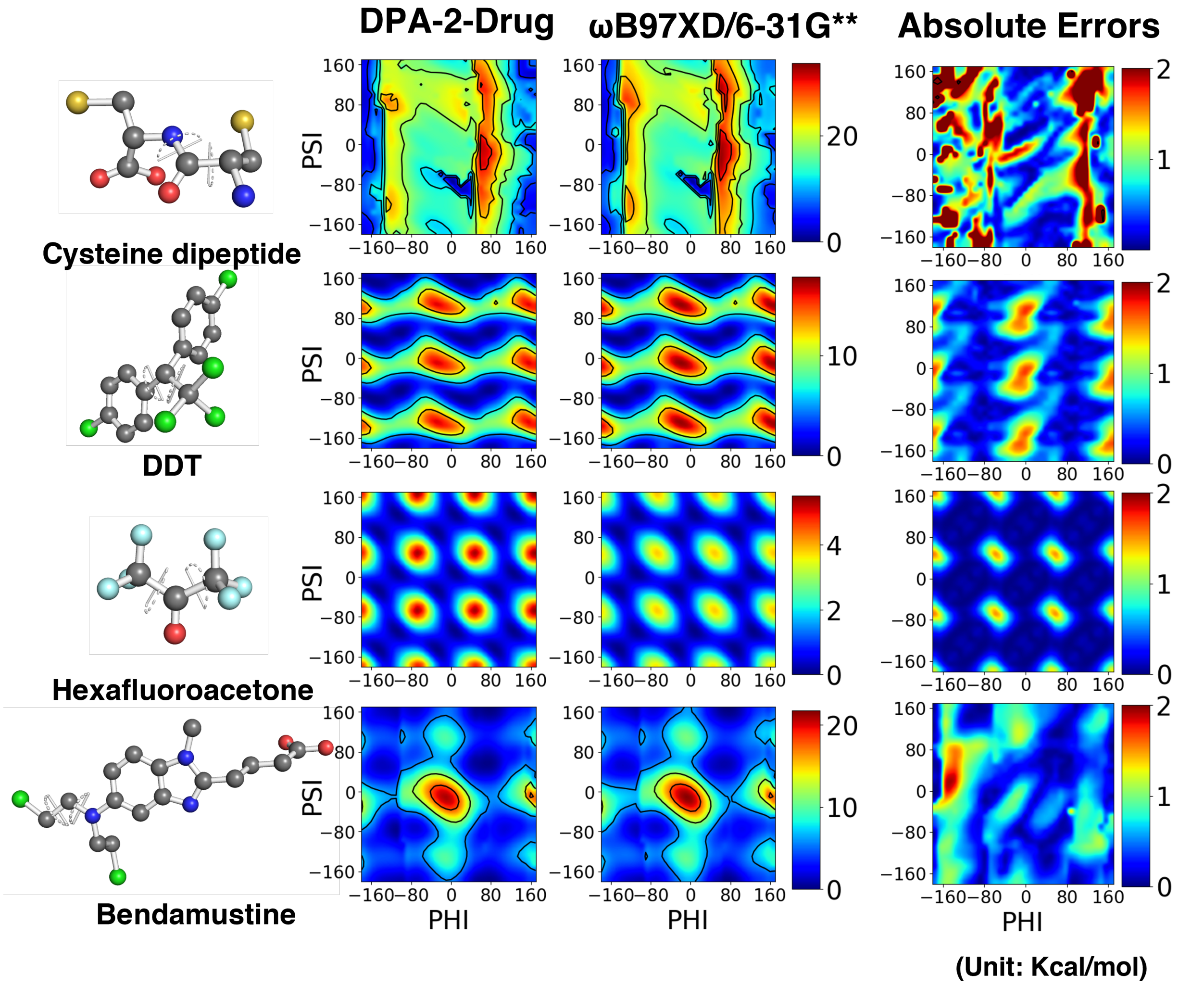


Figure S3: The relaxed 2D torsion profiles for four different molecules (cysteine dipeptide, DDT, hexafluoroacetone, and bendamustine) are shown for two optimization methods: DPA-2-Drug (left) and DFT ($\omega$B97XD/6-31G**; middle). The corresponding absolute errors between DPA-2-Drug and DFT are also provided (left). The scanned dihedral bonds are highlighted.

## References


(S1) Frisch, M. J.; Trucks, G. W.; Schlegel, H. B.; Scuseria, G. E.; Robb, M. A.; Cheeseman, J. R.; Scalmani, G.; Barone, V.; Petersson, G. A.; Nakatsuji, H.; Li, X.; Caricato, M.; Marenich, A. V.; Bloino, J.; Janesko, B. G.; Gomperts, R.; Mennucci, B.; Hratchian, H. P.; Ortiz, J. V.; Izmaylov, A. F.; Sonnenberg, J. L.; Williams-Young, D.; Ding, F.; Lipparini, F.; Egidi, F.; Goings, J.; Peng, B.; Petrone, A.; Henderson, T.; Ranasinghe, D.; Zakrzewski, V. G.; Gao, J.; Rega, N.; Zheng, G.; Liang, W.; Hada, M.; Ehara, M.; Toyota, K.; Fukuda, R.; Hasegawa, J.; Ishida, M.; Nakajima, T.; Honda, Y.; Kitao, O.; Nakai, H.; Vreven, T.; Throssell, K.; Montgomery, J. A., Jr.; Peralta, J. E.; Ogliaro, F.; Bearpark, M. J.; Heyd, J. J.; Brothers, E. N.; Kudin, K. N.; Staroverov, V. N.; Keith, T. A.; Kobayashi, R.; Normand, J.; Raghavachari, K.; Rendell, A. P.; Burant, J. C.; Iyengar, S. S.; Tomasi, J.; Cossi, M.; Millam, J. M.; Klene, M.; Adamo, C.; Cammi, R.; Ochterski, J. W.; Martin, R. L.; Morokuma, K.; Farkas, O.; Foresman, J. B.; Fox, D. J. Gaussian˜16 Revision C.01. 2016; Gaussian Inc. Wallingford CT.

(S2) Li, X.; Frisch, M. J. Energy-represented direct inversion in the iterative subspace within a hybrid geometry optimization method. *Journal of chemical theory and computation* **2006**, *2*, 835–839.

(S3) Wang, H.; Zhang, L.; Han, J.; E, W. DeePMD-kit: A deep learning package for many-body potential energy representation and molecular dynamics. *Computer Physics Communications* **2018**, *228*, 178–184.

(S4) Zeng, J.; Zhang, D.; Lu, D.; Mo, P.; Li, Z.; Chen, Y.; Rynik, M.; Huang, L.; Li, Z.; Shi, S.; Wang, Y.; Ye, H.; Tuo, P.; Yang, J.; Ding, Y.; Li, Y.; Tisi, D.; Zeng, Q.; Bao, H.; Xia, Y.; Huang, J.; Muraoka, K.; Wang, Y.; Chang, J.; Yuan, F.; Bore, S. L.; Cai, C.; Lin, Y.; Wang, B.; Xu, J.; Zhu, J.-X.; Luo, C.; Zhang, Y.; Goodall, R. E. A.; Liang, W.; Singh, A. K.; Yao, S.; Zhang, J.; Wentzcovitch, R.; Han, J.; Liu, J.; Jia, W.;

York, D. M.; E, W.; Car, R.; Zhang, L.; Wang, H. DeePMD-kit v2: A software package for deep potential models. *The Journal of Chemical Physics* **2023**, *159*, 054801.

(S5) Thompson, A. P.; Aktulga, H. M.; Berger, R.; Bolintineanu, D. S.; Brown, W. M.; Crozier, P. S.; in't Veld, P. J.; Kohlmeyer, A.; Moore, S. G.; Nguyen, T. D.; others LAMMPS-a flexible simulation tool for particle-based materials modeling at the atomic, meso, and continuum scales. *Comput. Phys. Commun.* **2022**, *271*, 108171.

(S6) Tribello, G. A.; Bonomi, M.; Branduardi, D.; Camilloni, C.; Bussi, G. PLUMED 2: New feathers for an old bird. *Computer physics communications* **2014**, *185*, 604–613.

(S7) Zhang, D.; Bi, H.; Dai, F.-Z.; Jiang, W.; Liu, X.; Zhang, L.; Wang, H. Pretraining of attention-based deep learning potential model for molecular simulation. *npj Computational Materials* **2024**, *10*, 94.

(S8) Zhang, D.; Liu, X.; Zhang, X.; Zhang, C.; Cai, C.; Bi, H.; Du, Y.; Qin, X.; Peng, A.; Huang, J.; others DPA-2: a large atomic model as a multi-task learner. *npj Computational Materials* **2024**, *10*, 293.

(S9) Devereux, C.; Smith, J. S.; Huddleston, K. K.; Barros, K.; Zubatyuk, R.; Isayev, O.; Roitberg, A. E. Extending the applicability of the ANI deep learning molecular potential to sulfur and halogens. *Journal of Chemical Theory and Computation* **2020**, *16*, 4192–4202.
